# A new species of *Myrmedonota* Cameron from eastern Kansas (Coleoptera, Staphylinidae, Aleocharinae)

**DOI:** 10.3897/zookeys.53.493

**Published:** 2010-08-27

**Authors:** K. Taro Eldridge

**Affiliations:** Department of Ecology and Evolutionary Biology, and Division of Entomology, Biodiversity Institute, 1501 Crestline Dr., Suite 140, University of Kansas, Lawrence, KS 66045-2811, USA

**Keywords:** Lomechusini, myrmecophily, termitophily, taxonomy, Nearctic region

## Abstract

Myrmedonota heliantha **sp. n.** is described from eastern Kansas (USA). All specimens were collected from dung. A modified new key to the species of Myrmedonota of America north of Mexico is provided.

## Introduction

The genus Myrmedonota Cameron (Aleocharinae: Lomechusini) currently contains 25 species, mostly described from the Old World (23 species), including Borneo (one species), Malay Peninsula (two species), New Guinea (19 species) and Sulawesi (one species) (Hlaváč personal communication). Recently, two new Nearctic species, Myrmedonota aidani Maruyama & Klimaszewski and Myrmedonota lewisi Maruyama & Klimaszewski were described from the states of Ohio and Indiana (USA), respectively (Maruyama et al. 2008). Life histories of most species are not known, but some have been collected in the presents of ants or termites; these species are presumed to be myrmecophilous or termitophilous, respectively (Bourguignon and Roisin 2006; Kistner 2003).

Recently, the author collected specimens of an undescribed Myrmedonota species from dung. This species is treated as new and described along with a new key to separate North American species.

## Methods

Dry specimens were observed using an Olympus SZX7 stereomicroscope. Dissected structures were observed with the stereomicroscope and an Olympus BX51 compound microscope. Illustrations were made using a camera lucida, Olympus U-DA, mounted on the compound scope. Scale bars were drawn using an Olympus slide micrometer. Body measurements were made using a stereomicroscope ocular micrometer.

All specimens examined were mounted using water soluble fish glue; all dissected body parts were cleared, preserved in Euparal mounting medium and are pinned under appropriate specimens.

Terminology follows the work of Gusarov (2002; aedeagal orientation), Maruyama (2006; epipharyngeal surface), Sawada (1970, 1972; chaetotaxy, mouthpart and median lobe morphology) and Seevers (1978; parameral morphology).

Abbreviations applied in this paper are as follows: HWhead width; HLhead length; HW/HLhead width over head length; OLocular length; OL/HLocular length over head length; CKTEprivate collection of K. Taro Eldredge; SEMCSnow Entomological Collection, University of Kansas.

## Systematics

### Genus Myrmedonota

Cameron, 1920

Myrmedonota See Kistner (2003) and Pace (2009) for references and keys to species.

#### Type species.

Myrmedonota cingulata Cameron, 1920, by monotypy (pp. 272–273).

#### Diagnosis.

Members of the genus Myrmedonota may be separated from other genera of Lomechusini by the following combination of characters (partially adopted from Maruyama et al. 2008): 1) head dorsally subcircular, excluding mouth parts; 2) head lacking neck; 3) occipital suture complete; 4) antennae generalized, clavate and slightly laterally compressed; 5) pronotum with complete marginal line; 6) pronotum without depressions or macrosculpture; 7) body surface finely punctate; 8) abdomen with no horn-like ornamentation; 9) dorsal abdominal surface with sparse to moderate setation but never with dense setal cover, nor with thick macrosetae creating a bristle-like texture; 10) cardo partially overlapping stipes, ventrally; 11) lacinia and galea extremely elongate and parallel sided; 12) labial palpomeres I and III subequal in length and longer then palpomere II; 13) glossa bifid with each lobe housing two sensillate elements; 14) mentum trapeziform and almost as long as wide; 15) labrum with lateral apices rounded and extending apically beyond maximum midpoint; 16) apical lobe of paramere short; 17) vellum and velar sac of paramere large and extending past the maximum reach of apical lobe and partially concealing it.

In North America Myrmedonota most closely resembles the genus Pella, but can be separated from the later by the following combination of characters: 1) smaller size (< 3.5 mm [Maruyama et al. 2008]); 2) extremely elongate lacinia and galea; 3) mentum almost as long as wide.

#### New key to Myrmedonota species of America north of Mexico

**Table:** 

1	Length of body at least 3.0 mm, ranging up to 3.2 mm; pronotum reddish brown or black in color; spermatheca with proximal end not curved atop itself (Fig. 12 & 21 in Maruyama et al. 2008)	2
–	Length of body less then 3.0 mm, on average 2.6 mm; pronotum yellowish in color; spermatheca with proximal end curved atop itself (Fig. 20)	Myrmedonota heliantha sp. n.
2	Pronotum reddish brown in color; elytra uni-colored reddish light brown; abdomen bi-colored with tergites II-IV light brown and V-VIII brown; spermatheca S-shaped (Fig. 12 in Maruyama et al. 2008); athetine bridge wide in lateral view (Fig. 8 in Maruyama et al. 2008)	Myrmedonota aidani Maruyama & Klimaszewski
–	Pronotum black in color; elytra bi-colored with at least distal margin cream colored and rest grey to black in color; abdomen uni-colored black; spermatheca V-shaped (Fig. 21 in Maruyama et al. 2008); athetine bridge narrow in lateral view (Fig. 17 in Maruyama et al. 2008)	Myrmedonota lewisi Maruyama & Klimaszewski

### Myrmedonota heliantha

Eldredge sp. n.

urn:lsid:zoobank.org:act:91552887-E437-4D63-88FB-80AAF8630A11

Figs 1 20 

#### Description.

**Figure 1–2.**
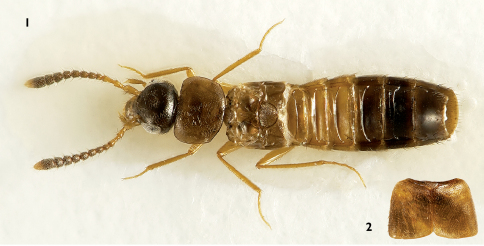
Myrmedonota heliantha. **1** habitus **2** elytra.

Body (Figs 1–2) length with a mean of 2.6 mm (n = 4), color yellowish to black. Head and abdominal tergites V-VII (segment V can be lighter or approaching yellowish grey) grey to black; pronotum and elytra yellowish light brown; abdominal tergites II–IV and VIII yellowish light brown to yellowish; mouthparts and legs yellowish; antennae dark brown, segments I–III and apex of segment XI may be yellow ish light brown to yellow ish brown.

**Figure 3–6.**
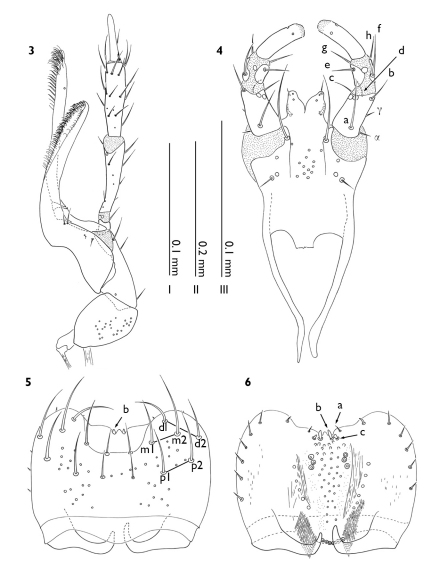
Myrmedonota heliantha. **3** right maxilla, ventral view **4** labium, ventral view **5** labrum **6** epipharynx. Scale bars: **I** Figs 5–6 **II** Fig. 3 **III** Fig. 4.

Head subcircular (HW = 0.47 mm; HL = 0.42 mm; HW/HL = 1.11; n = 4) with apex narrowing to receive labrum and mouthparts; eyes large, occupying half of head (OL = 0.21 mm; OL/HL = 0.5; n = 4); setae of vertex growing posteromedially; labrum (Fig. 5) with apex broadly margined, apicomedially with paired emargination to receive seta b; epipharynx (Fig. 6) with relatively short seta a, six to seven lateral setae equally spaced apart, mesolateral area with relatively little sculpture; maxilla (Fig. 3) with galea and lacinia extremely elongate, galea with preapical margin with a row of spinose setae that are uninterrupted by confused setation, palpomere IV long, with filamentous sensillae, and greater then half the length of palpomere III; labium (Fig. 4) with palpomere I and III subequal in length and palpomere II short, setula β and δ absent, glossa with a pair of apical and basolateral-epipharyngeal sensillate elements, mentum (Fig. 14) trapeziform with apex approximately half as wide as base and length almost equally width at base.

**Figure 7–14.**
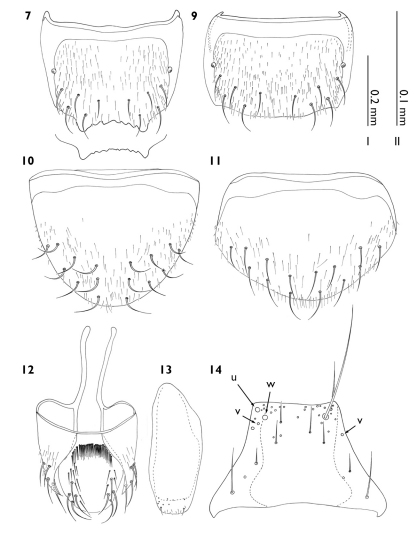
Myrmedonota heliantha male (7–9, 10–13) and female (10–11). **7** tergite VIII **8** holotype tergite VIII, outline of apical margin **9** tergite VIII **10** holotype sternite VIII **11** sternite VIII **12** genital segments excluding sternite IX, dorsal view **13** sternite IX, ventral view **14** mentum, ventral view. Scale bars: **I** Figs 7–13 **II** Fig. 14.

**Figure 15–20.**
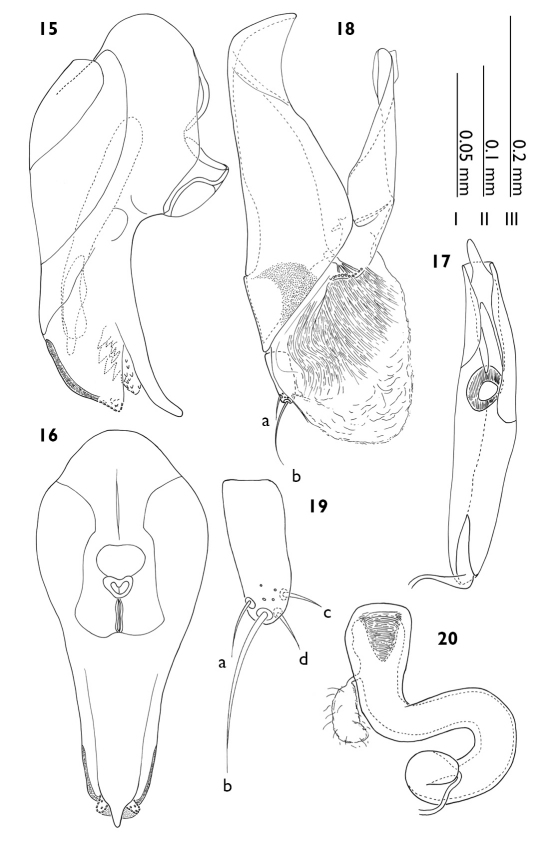
Myrmedonota heliantha. **15** median lobe, lateral view **16** median lobe, parameral view **17** copulatory piece, oblique arameral view **18** paramere, outer lateral view **19** apical lobe of paramere, outer lateral view **20** spermatheca. Scale bars: **I** Fig. 19 **II** Figs 17, 20 **III** Figs 15–16, 18.

Pronotum flattened, transverse (PW = 0.57 mm; PL = 0.39 mm; PW/PL = 1.46; n = 4), widest subapically and narrowest sub-basally, general form trapeziform (“approximately shield-shaped” [Klimaszewski et al. 2005, p. 709]), anterolateral corners rounded and slightly receding posterior for most of apical edge, posterolaterally with obtuse angles, posterior edge broadly rounded, lateral margins evenly arcuate, setae growing posterolaterally with no distinct midline.

Elytra together, transverse (EW = 0.73 mm; EL = 0.49 mm; EW/EL = 1.49; n = 2), longer then pronotum (EL/PL = 1.55) and acutely emarginated at suture.

Abdomen with dorsal surface relatively glabrous with tergites II-V with basal transverse impressions.

Male tergite VIII (Figs 7–8) apicomedially emarginate with lateral angles of emargination slightly produced and intermarginal edge variably serrate, with five pairs of macrosetae; sternite VIII (Fig. 10) with eight pairs of macrosetae; genital segments as in Fig. 12 and 13; aedeagus (Figs 15–16) relatively elongate with complete athetine bridge; copulatory piece (Fig. 17) long, occupying most of median lobe; paramere (Figs 18–19) with apical lobe partially visible on outer surface, internal velar pad present (stippled area), vellum large and extending apically, partially obscuring apical lobe in outer view, apical lobe with chaetotaxy as in Fig. 19.

Female tergite VIII (Fig. 9) truncate with five pairs of macrosetae; sternite VIII (Fig. 11) with seven pairs of macrosetae (one fewer then males); genital segments with same macrochaetotaxy as in males; spermatheca (Fig. 20) in shape of the letter S with proximal end curved atop itself, internal cone with circumventral sculptural grooves.

#### Diagnosis.

Myrmedonota heliantha most closely resembles Myrmedonota aidani, but can be distinguished by the following combination of characters: 1) pronotum widest subapically; 2) internal sac with distinctive configuration (Fig. 15); 3) spermatheca without an apical process extending from internal cone and with proximal end curved atop itself.

#### Material examined.

HOLOTYPE, ♂: “USA: KANSAS: Douglas Co.,/Lawrence, Baker Wetlands, N 38.92737°; W 95.23278°//ex. human dung baited pitfall trap (SEMC). PARATYPES, 2♀ and 1♂: same data as holotype (1♀, terminalia dissected but spermatheca not recovered, SEMC; 1♂, completely disarticulated permanent slide mount [additional label data “Euparal slide#007, K.T. Eldredge 2009”], CKTE); same locality data, differing data reads “13.ix.2009”, “ex. mammal dung” (1♀, terminalia dissected, SEMC).

#### Bionomics.

All specimens were collected off dung at Baker Wetlands, a 573 acre tract of restored wetland and prairie habitat, approximately 250 meters in elevation and two miles south of the University of Kansas campus.

#### Etymology.

Derived from the generic nomen Helianthus, in dedication to the sunflower state Kansas, where the type series was collected.

## Supplementary Material

XML Treatment for Myrmedonota

XML Treatment for Myrmedonota heliantha
